# The Incidence and Management of Pleural Injuries Occurring during Open Nephrectomy

**DOI:** 10.1155/2009/948906

**Published:** 2009-09-02

**Authors:** Ali Fuat Atmaca, Abdullah Erdem Canda, Ege Can Serefoglu, Serkan Altinova, Ahmet Tunc Ozdemir, M. Derya Balbay

**Affiliations:** Department of 1st Urology, Ankara Ataturk Training and Research Hospital, 06800 Ankara, Turkey

## Abstract

*Objective*. To evaluate the incidence, management, and risk factors of pleural injuries occurring during open nephrectomy. *Methods*. Between June 2004/and June 2008, 165 patients (167 renal units) underwent open simple (*n* = 37, 22.2%), partial (*n* = 39, 23.4%) or radical (*n* = 91, 54.5%) nephrectomy in our institution. *Results*. Flank, Chevron, and abdominal midline incisions were used in 148(88.6%), 17(10.2%), and in 2(1.2%) surgical procedures, respectively. Ribs were excised in 109(65.3%) procedures (11th rib, 10th-11th ribs, and 11th-12th ribs). Intraoperative pleural injuries were detected in 20(12%) procedures, 16(80%) were treated successfully with simple evacuation technique, and 4 required chest tube insertion. Age, sex, surgery type, incision type, and surgery site were not associated with pleural injury occurrence (*P* > .05). Rib resection was the only parameter associated with pleural injury occurrence. *Conclusion*. Pleural injuries occur in 12% of open nephrectomy procedures, and 80% can be repaired successfully. Few of them (2.4%) need chest tube insertion. Performing rib resection is a significant risk factor for pleural injury occurrence during nephrectomies.

## 1. Introduction

Due to the close anatomical relationship between kidneys and costadiaphragmatic recess of the pleural space, violation of the thorax might occur during performing open nephrectomy. It has been reported that rib resection might increase the risk of pleural injury via flank incision [1, 2]. Pleural injuries that occur during performing nephrectomy can be diagnosed easily and can be repaired successfully by simple evacuation technique [2, 3]. However, a small percent of these patients might require postoperative chest tube insertion due to the presence of manifest pneumothorax although repaired intraoperatively [3]. Incidence of pneumothorax after open nephrectomy is reported to be between 1–10% [1]. Nephrectomies performed via anterior tranperitoneal subcostal incisions have a decreased pleural injury occurrence risk compared to flank incisions [1].

In our study, we investigated the incidence, risk factors, and management of pleural injuries that occur during simple, partial, or radical nephrectomy performed via abdominal midline, Chevron, or flank incisions with or without rib resection. 

## 2. Materials and Methods

Between June 2004 and June 2008, 165 patients including 167 renal units underwent open nephrectomy via abdominal midline, Chevron, or flank incisions in our institution. Open simple nephrectomy was performed in 37 kidneys (22.2%), partial nephrectomy (PN) was performed in 39 kidneys (23.4%), and radical nephrectomy (RN) was performed in 91 kidneys (54.5%). Indications for open simple neprectomy were nonfunctioning kidney associated with hydronephrosis or pyonephrosis in 29 patients, nonfunctioning kidney associated with renal hypoplasia in 5 patients, traumatic kidney laceration in 2 patients, and renal cyst hydatic in 1 patient. Indications for open PN were renal mass lesions in 37 patients. One patient with bilateral nonfunctioning lower kidney pole moieties which developed due to hydronephrosis with stones having bilateral duplicated collecting systems underwent bilateral PN (totally 39 kidney). Indications for RN were renal mass lesions in all patients. One patient underwent RN and PN in different sessions via flank incisions, and 11th ribs were removed in each surgical procedure. 

Intraoperatively, evidence for pleural opening was investigated in all patients by performing water seal test. If present, pleural opening was repaired by using a running 3/0 polyglycolic suture after simple evacuation of the air present in the pleural space. The water seal test is performed by filling the surgical area including the diaphragmatic and pleural neighboring sites with sterile saline up to the level of the skin incision and observing presence of any air bubbles coming through the pleura and/or diaphragmatic structures during respiration of the patient.

Chest X-rays for the detection of pneumothorax were obtained in the postanesthesia care unit in all patients who underwent intraoperative pleural repair. Chest tube is inserted if manifest pneumothorax is detected in the chest X-ray. 

Factors that might affect formation of intraoperative pleural injury such as age, gender, site of surgery, type of surgery, type of incision, and presence of rib resection(s) were investigated. Patient charts were used in order to obtain these data.

Continuous variables were compared using the Student's *t*-test. Categorical variables were compared using chi-square and Fisher's exact tests. Logistic regression analysis was used in order to identify variables that were asssociated with pleural injury. Significance level of *P*-value was set .05. All statistical analyses were performed by using SPSS version 13.0. 

## 3. Results

Patient and surgery characteristics are summarized on Table 2. Intraoperative pleural injuries were detected in 20 (12%) surgical procedures. Of these, 16 (80%) were treated successfully with simple evacuation technique. Postoperative chest X-rays demonstrated manifest pneumothrax in 4 (20%) patients who required chest tube insertion (overall 2.4%) due to unsuccessful repair. 

Table 1 represents analysis of risk factors associated with intraoperative pleural injury occurrence. Among the factors that were investigated, performing rib resection was the only factor which was significantly associated with pleural injury occurrence during nephrectomy. When patients with flank incisions were evaluated, of the 109 procedures with rib resections, pleural injuries occurred in 18 cases (16.5%). However, of the 39 procedures without rib resections, pleural injury occurred only in 1 case (2.6%) (*P* = .025). Logistic regression was used in order to calculate the odd's ratio (OR). OR was calculated as 5.54 (*P* = .025, 95% CI : 1.24–24.78). 

Although no intraoperative pleural injury was detected, subcutaneous emphysema developed in the postoperative followup in a patient with RN which was performed via flank incision including resection of both 10th and 11th ribs that subsided within 3 days with conservative management. 

## 4. Discussion

Intraoperative pleural injuries can be detected easily. However, in order not to miss small pleural injuries that might occur during surgery, the operation area could be filled with sterile saline and the anesthesiologist could be asked to hyperinflate the lungs which would lead to the formation of air bubbles as a sign of pleural opening. We routinely perform this test in all nephrectomies in order to diagnose the presence of possible pleural injuries. If pleural injury is detected then we insert a small caliber catheter through this pleural opening into the chest cavity and we submerge the free end of the catheter into a container full of sterile saline solution. We use an absorbable running suture such as 3/0 polyglycolic acid suture around this catheter to close the pleural opening. We again ask the anesthesiologist to perform positive ventilation until the air bubbling in the container disappears which means that all the air present in the chest cavity due to the pleural injury is drained. Then, the 3/0 polyglycolic acid suture is tied as the catheter is removed simultaneously [2, 4]. When the patient is taken to the postanesthesia care unit after the surgery, we ask for a chest X-ray to be performed in order to see if any manifest pneumothorax is present. If pneumothorax is detected on chest X-ray then, we insert a chest tube.

In a large single center study, Stephenson et al. reported postoperative pneumothorax incidence as 1.1% after PN (4 in 361 cases) and 0.9% after RN (6 in 688 cases) [5]. All of 4 the patients with pneumothorax after PN and 4 out of 6 patients with pneumothorax after RN required chest tube insertion in their series. In a population-based sample, Joudi et al. reported iatrogenic pneumothorax incidence as 5.5% after PN and 4.9% after total nephrectomies [6]. However, none of these two studies reports any data concerning the type of incisions used and any rib resections performed or if any pleural injuries were detected and repaired intraoperatively. 

The risk of pleural injury is increased in flank incisions if rib resection is also performed [2, 4]. It was reported that patient age and site of the surgical procedure do not affect pleural injury occurrence during surgery [2–4]. Although some studies suggested that gender does not have an impact on intraoperative pleural injury development [3, 4], Poore et al. reported that its incidence is significantly increased in female patients [2]. In our study, we did not detect any significant association between patient age, gender, type of surgery, or site of surgery and pleural injury occurrence (Table 1). Additionally, we did not observe significant association between the type of incision and pleural injury occurrence; however, the number of patients in the Chevron and midline incision groups is limited in our study. The only parameter that was significantly associated with pleural injury occurrence was performing rib resection (Table 1). When we compared nephrectomies via flank incisions with or without rib resections, performing rib resection was found to be a significant risk factor for pleural injury occurrence regardless from the type and site of surgery performed (*P* < .05). 

Performing routine chest X-ray is controversial after surgeries which have an increased risk of pleural injury occurrence such as nephrectomies. We recently demonstrated that routine chest X-ray is not necessary if intraoperative pleural injury is not detected in patients who were operated via flank incisions with rib resections [3]. Similarly, others also showed that routine chest X-ray is not necessary in these patients and does not change patient management [1, 2, 4]. 

Another important issue in patients with intraoperative pleural injuries is to insert prophylactic chest tube routinely in addition to intraoperative pleural injury repair. It has been suggested that if the intrapleural rent is repaired adequately during surgery, the incidence of postoperative pneumothorax necessitating chest tube insertion is very low [2–4, 7]. Due to our surgical experience, only 4 patients (2.4%) required postoperative chest tube insertion among 20 patients (12%) whom we performed pleural injury repairs which support the published literature. If we performed chest tube insertion in all patients with intraoperative pleural injuries, in 80% of these patients (overall 9.8%) this procedure would have been unnecessary. Besides, if we did not repair all of the pleural injuries intraoperatively, it would have been difficult to know to what extent these would cause clinically significant pneumothorax. In addition, complications like pneumonia and development of atelectasis are reported to be seen less with associated lower pain scores and shorter length of hospital stays in patients whose pleural injuries are repaired intraoperatively without chest tube insertion [7]. Therefore, we do not recommend inserting prophylactic chest tube in these patients in order to avoid complications related with chest tube, and to decrease patient costs, shorten length of hospital stay and increase quality of life. 

## 5. Conclusion

Performing rib resection seems to be the most significant risk factor associated with pleural injury occurrence during open nephrectomies. Including surgeries via flank incisions, pleural injury occurrence risk is quiet low if rib resection is not performed. Intraoperatively, pleural injuries can be diagnosed and repaired easily with a success rate of 80%. We suggest performing intraoperative water seal test in all patients who underwent open nephrectomy irrespective of the incision used and if pleural injury is present we recommend repairing it by simple evacuation technique using a running 3/0 polyglycolic suture without chest tube insertion. We also suggest performing an immediate postoperative chest X-ray in order to see if manifest pneumothorax is present in these patients and if present we recommend chest tube insertion. 

## Figures and Tables

**Table 1:** Analysis of risk factors for occurrence of pleural injury during nephrectomy.

Pleural injury	(+)	(−)	*P*-value
Age	58.1 ± 12.1	53.8 ± 15.1	.221
Gender: *n*, (%)			
* *Male	11, (10.5)	94, (89.5)	.437
* *Female	9, (14.5)	53, (85.5)	
Side: *n*, (%)			
* *Right	10, (11.2)	79, (88.8)	.753
* *Left	10, (12.8)	68, (87.2)	
Type of surgery: *n*, (%)			
* *SN	1, (2.7)	36, (97.3)	
* *PN	4, (10.3)	35, (89.7)	.087
* *RN	15, (16.5)	76, (83.5)	
Rib resection: *n*, (%)			
* *Yes	18, (16.5)	91, (83.5)	.013
* *No	2, (3.4)	56, (96.6)	
Incision: *n*, (%)			
* *Flank	19, (12.8)	129, (87.2)	.475
* *Chevron and midline	1, (5.3)	18, (94.7)	

SN: simple nephrectomy, PN: partial nephrectomy, RN: radical nephrectomy.

**Table 2:** Characteristics of patients included in our study.

Number of patients	165
Number of nephrectomies	167
Age	
* *Mean	54.3 ± 14.9
* * Median	56
* *Range	9–82
Gender: *n*, (%)	
* *Male	104, (63)
* *Female	61, (37)
Nephrectomy side: n, (%)	
* *Right	89, (53.3)
* *Left	78, (46.7)
Type of surgery: *n*, (%)	
* *SN	37, (22.2)
* *PN	39, (23.4)
* *RN	91, (54.5)
Rib resection: *n*, (%)	
* *No	58, (34.7)
* *Yes	109, (65.3)
* * * *11th	100, (91.7)
* * * *10th and 11th	4, (3.7)
* * * *11th and 12th	5, (4.6)
Type of incision: *n*, (%)	
* *Flank	148, (88.6)
* *Chevron	17, (10.2)
* *Midline	2, (1.2)
Pleural injury: *n*, (%)	
* *No	147, (88)
* *Yes	20, (12)
Chest tube insertion: *n*, (%)	
* *No	16, (80)
* *Yes	4, (20)

SN: simple nephrectomy, PN: partial nephrectomy, RN: radical nephrectomy.
